# An automated method for large-scale monitoring of seed dispersal by ants

**DOI:** 10.1038/srep40143

**Published:** 2017-01-10

**Authors:** Audrey Bologna, Etienne Toffin, Claire Detrain, Alexandre Campo

**Affiliations:** 1 Unit of Social Ecology, Université Libre de Bruxelles, Campus de la Plaine, Brussels, Belgium

## Abstract

Myrmecochory is the process of seed dispersal by ants; however, it is highly challenging to study, mainly because of the small size of both partners and the comparatively large range of dispersal. The mutualistic interaction between ants and seeds involves the former retrieving diaspores, consuming their elaiosome (a nutrient-rich appendage), and the rejection of seeds from the nest. Here, we introduce a semi-automated method based on stitching high resolution images together, allowing the study of myrmecochory in a controlled environment over time. We validate the effectiveness of our method in detecting and discriminating seeds and ants. We show that the number of retrieved diaspores varies highly among colonies, and is independent of both their size and activity level, even though the dynamics of diaspore collection are correlated with the arrival of ants at the food source. We find that all retrieved seeds are rejected from the nest in a clustered pattern, and, surprisingly, they are also frequently redispersed within the arena afterwards, despite lacking elaiosome. This finding suggests that the dispersal pattern might be more complex and dynamic than expected. Our method unveils new insights on the mechanisms of myrmecochory, and could be usefully adapted to study other dispersal phenomena.

Ants are ubiquitous insects that have developed many mutualistic interactions with various types of organisms, including plants (e.g. *Acacia*-*Pseudomyrmex* interaction^1^) and other insects, like hemipterans that produce honeydew^2^ (e.g. aphids^3^, or mealybugs^4^), in addition to fungi and bacteria (leaf-cutter ants^5^). Myrmecochory is an ant-plant interaction, in which ants manipulate a particular kind of diaspore bearing a nutrient-rich appendage called an elaiosome. This process has three steps: (1) ants collect diaspores and bring them back to the nest (e.g. refs 6 and 7), where (2) the workers and, mainly, larvae eat the elaiosomes^6^,^8^,^9^,^10^, after which (3) the seeds are rejected from the nest^7^,^11^,^12^. Indeed, as myrmecochorous seeds primarily depend on carnivorous and omnivorous ants for dispersal; consequently, seeds lacking elaiosome are of no interest to these ants^13^,^14^,^15^.

Seed dispersal by ants is viewed as a diffuse mutualism, in which each partner (ant and diaspore) interacts with one or more species of the other partner^16^,^17^. For ants, consumption of the elaiosome might be nutritionally beneficial^9^,^18^,^19^,^20^,^21^,^22^, because it is rich in proteins, amino acids, carbohydrates and fatty acids. In fact, the elaiosome has been compared to a dead insect in terms of fatty acids content^15^. For plants, many benefits accompany seed dispersal, such as their deposition in nutrient-enriched microsites, facilitation of their germination^23^, predator and fire avoidance^24^, breaking of dormancy^25^, and reduction of parent-offspring and seedling-seedling competition^26^,^27^.

Myrmecochorous plants have various seed releasing strategies^28^,^29^,^30^,^31^,^32^ that directly determine the pattern of seed dispersal on the ground, particularly with respect to quantity and density, which influence the seed harvesting behaviour of ants^7^,^11^,^33^,^34^,^35^. Other factors that influence harvesting behaviour include plant phenology, the size of the elaiosome^12^,^36^,^37^,^38^, the elaiosome/seed size ratio^39^,^40^, and the chemical composition of the elaiosome^15^,^41^,^42^,^43^. In the presence of large food resources, some ant species are able to elicit recruitment^44^. However, to date, this process has only been rarely observed in the context of myrmecochorous diaspore foraging^45^,^46^. Because diaspores are separate items, it is generally assumed that ants do not need a recruitment process for their collection.

Studies about seed dispersal based on myrmecochory are usually restricted to the transportation of diaspores to ant nests, without considering their ultimate rejection from the nest, or just their removal to nest mounds. To our knowledge, only a few studies have highlighted the rejection of seeds from ant nests, using vague terms to label this process, including “seed relocation” and “seed redispersal”^7^,^11^,^33^,^34^,^47^,^48^,^49^,^50^,^51^,^52^,^53^,^54^. The time that diaspores are retained inside the nest and the distance to which they are dispersed after rejection from the nest seem to be species- and ecosystem-specific. However, in European temperate areas, the typical seed rejection distance from the nest is about 1–1.5 m^11^,^12^,^34^.

The rejection of seeds from ant nest makes them available for potential redispersal (also called secondary dispersal). This process could strongly influence their ultimate spatial pattern and, thus, the final distance reached by seeds^51^. The lack of knowledge about dispersal distances and about rejection/redispersal phenomena is mainly assigned to the methodological difficulty in detecting seeds of a few millimetres in length in the field, preventing the acquisition of quantitative data. However, two labelling methods have been used in the field: a method using a magnetized coded wire tag inserted inside the seeds^50^ and another method combining painting and radiolabeling^48^. Both methods have their own advantages and weaknesses; however, their common limitation is the fact that they change the inherent nature of diaspores and do not allow the continuous monitoring of seed dispersal patterns.

To advance our understanding of myrmecochory, we must monitor how ants forage for diaspores and how they later distribute the seeds in their environment as precisely as possible and throughout the same experiment. Here we introduce a novel, semi-automated method, based on stitching large resolution images together, to detect seeds (of millimetre scale) in a controlled environment of more than 12 m^2^. The high resolution images facilitate the detection of individual seeds and ants, with a large scale ratio between these items (seeds and ants) and the surveyed arena. As a result, we are able to employ image-processing techniques to locate the seeds in the experimental setup during both foraging and dispersal, as well as the distribution of workers over the entire arena. The combination of image stitching and seed detection allows monitoring of ants’ activity in detail. As a consequence, we expect to contribute new insights about the spatio-temporal patterns of seed following rejection from the nest.

## Material and Methods

### Plant species

The great celandine (*Chelidonium majus*, Linnaeus, 1753) is a perennial herbaceous plant. This nitrophilous species grows in disturbed areas like rock remains or old walls. It is blooming from April to October^55^ with two production peaks in early spring and autumn. *C. majus* is a diplochorous species, meaning that seeds are first released balistically over the ground before being harvested by ants. Seeds are black-brown of about 2 mm in length and about 1.5 mm in width and their elaiosomes are fleshy and soft, of about 1.5 mm in length and 0.8 mm in width. Mature diaspores of *C. majus* were collected in June 2013 on one site in Udange, Belgium. They were immediately stored at −20 °C until their use for experiments, a storage procedure that does not seem to affect behaviour of ants towards the diaspores^36^.

### Ant species

The red ant (*Myrmica rubra*, Linnaeus 1758), is a temperate species whose colonies are composed of hundreds of workers of 4–6 mm length^56^. This ant species is mainly carnivorous but also feeds on sugar sources such as honeydew and plant nectaries^57^. It is also well known for retrieving elaiosome-bearing seeds from myrmecochorous plants^9^,^21^,^36^.

The twelve ant colonies were collected during summer of 2014 on three different sites in Belgium. They contained brood, 200–400 individuals, as well as 3–6 queens. Colonies were reared in the laboratory into detachable nests consisting of two glass plates superimposed (15 × 15 × 0.4 cm) and separated by microscope glass slides used as walls. The upper glass plate was perforated in its centre with a hole (diameter =1.5 cm). Nests were moistened once a day by pouring water on blotting paper covering the floor glass plates. Colonies were kept at room temperature of 20 °C–22 °C, at a relative humidity of 55 to 65% and a constant photoperiod of 14 h day and 10 h night. Ants were supplied with sucrose solution (0.3 M) *ad libitum* and one mealworm (*Tenebrio molitor*) three times a week.

### Experimental setup and procedure

**Figure 1:**
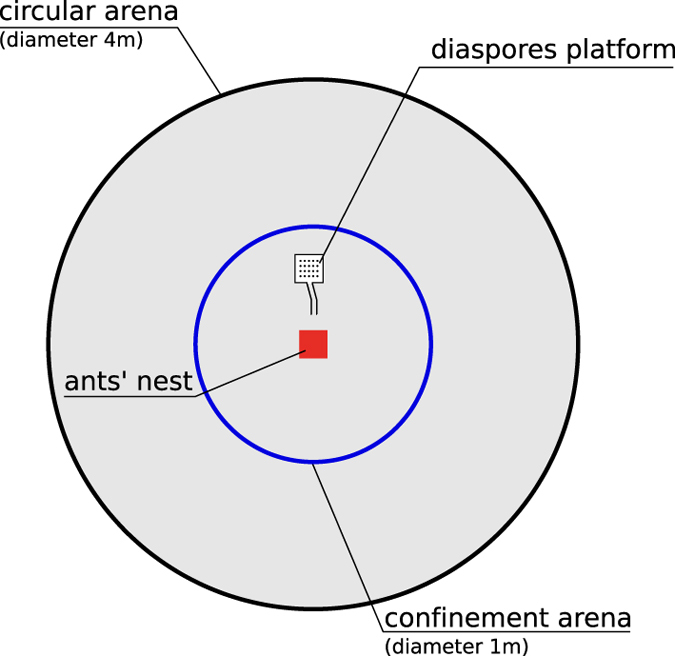
Sketch of the experimental setup as seen from above.

Each colony was starved during four days prior experiments. On the 4^*th*^ day, the removable nest was placed in the centre of a large circular arena of 4 m diameter with a fluon-coated wall (Fig. 1).

**Figure 2:**
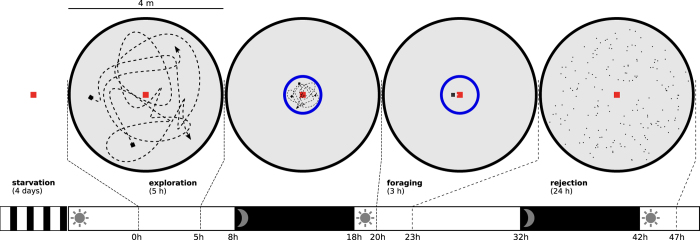
Timeline of events. Nest is depicted by the red square, while blue circle shows the confinement arena. Photoperiod is represented by the white (day) and black (night) rectangles at the bottom of the figure.

Each experiment was broken down into three main stages (Fig. 2): exploration of the arena, diaspore foraging, and seed rejection. During the exploration stage, ants were left free to wander in the arena so that they got accustomed to their new environment during 5 hours. After that, exploring foragers were collected and put back into the nest, while their exploration range was restricted with a confinement-arena overnight (1 m diameter arena centred on the nest).

The foraging stage occured the following day: a platform (10 cm × 10 cm) containing 200 diaspores was placed in front of the nest, and could be accessed via a bridge (length = 30 cm; width = 1 cm). The diaspores were located at least 1 cm from the edge of the platform and were separated from each other by 8 mm. During this stage, the confinement arena restricted workers’ motion to speed up discovery of diaspores. The foraging stage lasted 3 hours after which the platform and the confinement arena were removed.

The rejection stage spanned over 24 hours, during which the collected seeds were rejected out of the ants’ nest. The final number of seeds released in the arena at the end of the rejection stage was counted by hand and the colony removed from the arena and brought back into rearing nests. The photoperiod was preserved during the experiment, leading to a 10 hours dark period during which observation of the seeds’ rejection within the arena was not possible (Fig. 2).

### Data acquisition and extraction

Using our experimental protocol, we aimed at characterizing the behaviour of ants as well as at facilitating investigation about the spatio-temporal pattern of rejected seeds. Also, to minimize external disturbances, we automatized data acquisition as much as possible, while reducing any human intervention to the minimum. The entire workflow of data extraction from pictures and movies was performed with the USE Tracker free software^58^. This software offers a range of image processing algorithms that can be parametrized and later combined in any sequence through a pipeline. USE Tracker relies on various state of the art, free software libraries including FFMPEG for decoding and encoding videos, OpenCV^59^ for image processing algorithms, and WxWidgets for the graphical interface.

### Arena

Within the arena, the large ratio of dispersal distances over seeds size (2000:1) required large images of the arena with a high pixel density to detect seeds’ automatically and reliably.

#### Image stitching

**Figure 3:**
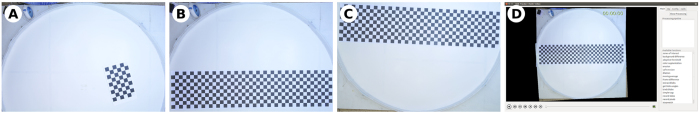
Calibration and stitching of source images to produce a single large resolution image. (**A**) Each camera is calibrated using a series of images that contain a checkerboard in random positions. (**B** and **C**) Top and bottom view of the arena. Both images overlap, and a large checkerboard lies in the overlapping region. The checkerboard is recognized by USE Tracker in both pictures and used to calculate corresponding coordinates. (**D**) After applying the stitching algorithm, the result is a single, large resolution image of the entire arena.

Image stitching is a technique commonly used in modern digital applications to produce panoramas. It consists of compositing several pictures together so as to produce a new one, with increased dimensions. If the images cover different parts of the surface to observe, then stitching these images into a single one allows to increase the image resolution. As shown in Fig. 3, we stitched together high resolution images to be able to detect 2 mm seeds anywhere in the arena.

Original pictures of the circular arena were captured using two DSLR (Nikon D5100, lens 100 mm, and a Nikon D5200, lens 100 mm), which offer excellent image resolution for a reasonable price. Because we had two different cameras, images differed in their dimensions. The Nikon D5100 outputs images of 4928 pixels × 3266 pixels, while the D5200 outputs images with a resolution of 6000 pixels × 4000 pixels. The cameras were remotely controlled by a main computer using gphoto2 software^60^. With this open source software, pictures were grabbed simultaneously every 10 minutes and immediately transferred to a storage hard drive. The distortion of each camera lens was measured before the experiments, and calibration parameters were used to produce corrected images^61^,^62^. The images captured by both cameras have an overlapping region. To find the correspondence between the two images, we placed a calibration checkerboard in the overlapping region (Fig. 3B and C) and determined the optimal transformation matrix (known as the homography). This matrix takes the coordinates of an object in one image and returns the coordinates of the same object in the other picture.

The stitching algorithm is based on common algorithms found in the literature, and implemented using OpenCV free software^59^, but it differs in the way images are combined. Usually, images are blended to produce a visually appealing result, with no visible discrepancies to the human eye. However, this technique can introduce artefacts in which the same graphical elements can appear twice or blurred. For seed detection, we prefered to not blend images but simply define a stitch line, which is a border that delimits where pixels originate from. In this way, we combined images without blending overlapping regions (see Fig. 3). Moreover, because of noise and hardware differences between cameras, images can have a slightly different exposure which make image processing more difficult, requiring different parameters for each regions. We dealt with this problem early in the processing, by calculating the average luminosity of all the captured images and then compensating their luminosity to match the global average. Once image exposure was corrected, we applied the transformation matrix to combine the pixels taken from both images and to express them in the same coordinate system. The result, as seen in Fig. 3D was a single large image (6170 pixels × 6246 pixels, or 38.5 megapixels) with homogeneous exposure, no blending artefacts, as if taken by a very large resolution camera.

#### Image analysis

**Figure 4:**
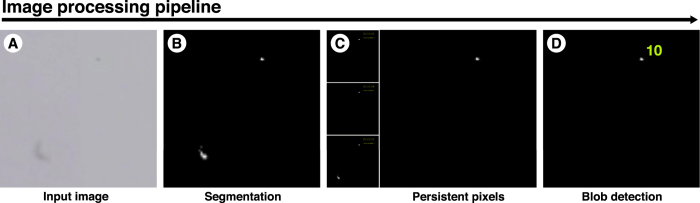
The different steps of the image processing pipeline in details. (**A**) Zoom over a limited portion of the arena, showing a seed and the blurry trace of an ant. (**B**) Resulting detection from background difference and colour segmentation, the seed and the ant are both detected. (**C**) Resulting detection from the persistent pixels algorithm. The three previous frames are used, only the pixels present in at last 2 frames are retained. This eliminates the trace of the ant. (**D**) The blob detection algorithm recognizes the seed as a group of 10 contiguous pixels.

For each experiment, we processed our high resolution images with the sequence of algorithms depicted in Fig. 4. First, we calculated a static background image, which is a picture of the experimental setup that does not contain any seed or ant. The background image was obtained by analysing 15 captured images from the experiment and retaining for each pixel the most represented red-green-blue (RGB) triplet. Possible artefacts, traces of ants or seeds were removed manually. Then, we calculated the difference between each image and the background image (Fig. 4B), and used segmentation to identify pixels that showed a significant difference, i.e. above a defined threshold value. In practice, this step highlighted areas where an ant or a seed was, but noise in either image could leave false positives. Hence we refined our selection by applying a second segmentation based on colour, retaining only the darkest pixels.

Once all the pixels covered by ants or seeds were detected, we needed to discriminate them in order to focus on the location of seeds. Considering that ants are active and often moving, and that seeds dropped on the ground remain static for longer periods of time, we relied on the persistence of pixels to decide whether they represent ants or seeds. To do so, we used an algorithm that only retains pixels that are present in at least two out of three consecutive images (Fig. 4C). Finally, remaining false positive (i.e. static ants) were discriminated from seeds based on their size using a blob detection algorithm (Fig. 4D). This algorithm pools contiguous pixels into entities called blobs, which correspond to physical entities such as ants or seeds, and keeps or discards them based on minimum and maximum threshold blob size in pixels. With this algorithm, individual ants within the arena were identified (no threshold size), while seeds were unambiguously discriminated from ants using their typical size ranging from 5 to 19 pixels (corresponding to rounded seeds of width comprised between 1 and 2.5 mm, on images with resolution of 0.677 mm/pixel). Extracted data consisted of the cartesian coordinates and the size in pixels of ants and seeds.

### Foraging platform

During the foraging stage, we recorded the ants reaching and leaving the platform as well as the diaspores still present there using a CCTV camera (Panasonic WV-BP330). From the video recordings, we extracted both the incoming and outgoing flow of ants using the counting plugin of USE Tracker. The number of diaspores *D*_*track*_ present on the platform was determined in USE Tracker using a segmentation algorithm to detect dark pixels on the platform. Subsequently, we subtracted the number of ants located on the platform to obtain the number of remaining diaspores. For validation purposes, we used *D*_*count*_, which represents the number of diaspores on the platform obtained by manual counting. (see Supplementary: S1 Automatic census of remaining diaspores on the platform).

Due to a hardware deficiency, 3 videos of the foraging platform were lost out of the 12 replicates. Hence, our analysis of the collection dynamics of diaspores was restricted to 9 replicates.

### Data analysis

A summary of extracted raw data and variables used for the analysis is given in Supplementary (see Table S1). All statistical analysis of the data were conducted using R software version 3.2.2^63^, with a significance level for all statistical tests set to *α* = 0.05. When normality of the distribution was confirmed (Shapiro-Wilk test), results were presented as mean ± sd (n), while medians [Q1; Q3] (n) were used when the data does not follow a Gaussian distribution.

Distances from the nest and angles of seeds within the arena were computed from their cartesian coordinates. Circularity of the seeds distribution within the arena was assessed using Rao’s spacing, Kuiper, Watson, and Rayleigh tests from R CircStats package^64^.

To characterize the spatial pattern of rejected seeds, we used the *G(d*) function, which is the cumulative fraction of the seeds nearest neighbour distance (*NNdist*) as a function of the considered distance *d*^65^. First, we computed the experimental *D(d*) curve at the end of a replicate. Then we simulated the random dispersal of the same number of seeds following a Poisson process within a defined area of interest containing the seeds (Fig. 8B). Such simulation process generates a pattern of seeds referred to as Complete Spatial Randomness (CSR). We simulated a total of 1000 CSR patterns and compute their *D(d*) function to define a pointwise envelope (Fig. 8C). Then, we compared the location of the experimental *D(d*) curve with the envelope of simulations to characterize the spatial pattern observed. If the experimental *D(d*) curve lies within the pointwise envelope, it indicates that the experimental pattern follows a CSR. On the contrary, the departure of the experimental *D(d*) curve above the pointwise envelope indicates a high proportion of short inter-seed distances characteristic of a clustered pattern, while a departure below the envelope indicates a regular pattern. We tested the statistical significance of this departure with the Diggle Cressie Lossmore Ford (DCLF) test, with one-sided upper deviation alternative hypothesis. We limited the possible bias due to the border of the arena by defining the area of interest as the portion of arena that was at least 5 cm away from its border. Only the seeds located in the area of interest were considered for the analysis and the simulations of CSR. When patterns were characterized as clustered, the fraction of nearest neighbour distances lying above the envelope was referred to as characteristic clustering distance *NNdist*_*cluster*_ (Fig. 8C), while the same fraction of nearest neighbour distances from the associated 1000 simulated CSR patterns was referred to as characteristic CSR distance *NNdist*_*CSR*_. All these analyses were realised using R Spatstat package^66^.

## Results

### Validation of the method

**Figure 5:**
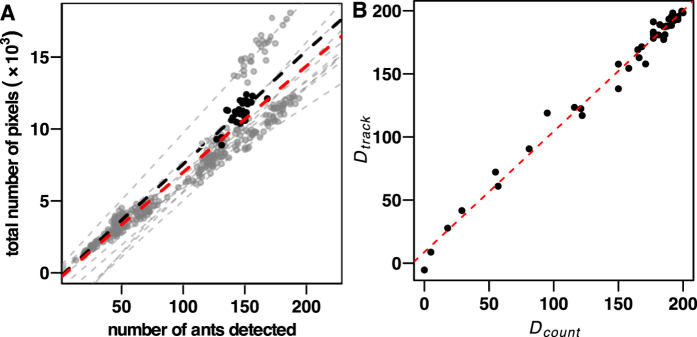
The automated detection method is effective at detecting items at each stage of the myrmecochory process. (**A**) During the exploration stage, effectiveness of ants detection was relatively stable whatever the number of ants in the arena, each replicate showing a statistically significant linear relationship between the number of ants detected and the total number of corresponding pixels. Similar regression slopes for the different replicates indicated that detection effectiveness was constant for our entire experimental pool. Black dots and black dotted lines stand for values and linear regression of replicate 2, while grey dotted lines indicate global regression line for each replicates and red dotted line shows common regression line for all replicates (linear relationship: *N*_*pixels*_ = 73.6 *N*_*ants*_ − 346.4, *R*^2^ = 0.87, *F*_1,345_ = 2.39 × 10^3^, *P* < 0.0001). The same tendency was observed during foraging stage (see Supplementary Fig. S3). (**B**) The good agreement between manual counting (*D*_*count*_) and automatic detection (*D*_*track*_) of retrieved diaspores showed the reliability of the diaspores tracking (linear relationship: *D*_*track*_ = 0.96 *D*_*count*_ + 8.80, *R*^2^ = 0.99, *F*_1,43_ = 3.02 × 10^3^, *P* < 0.0001).

Effectiveness and consistency of ants’ detection by USE Tracker were assessed by determining the relationship between the number of ants (i.e. blobs) detected on the arena and the corresponding total amount of pixels at each frame, for both exploration and foraging stages. There was a significant linear relationship between these values (*P* < 0.001 in each stage and each replicate) indicating that the detection sensitivity remained relatively stable whatever the number of workers detected, either during exploration (*R*^2^ = 0.69 ± 0.15, n = 12; Fig. 5A) or foraging stages (*R*^2^ = 0.82 ± 0.08, n = 12; see Supplementary Fig. S3). Moreover, a comparison of regression slopes indicated that the different replicates were not statistically different, whatever the stage considered (see Supplementary Tables S2 and S3). Hence, USE Tracker consistently detected ants, whatever their number in the arena. This confirmed the robustness of our detection method throughout our entire set of replicates.

During the foraging stage, the effectiveness of automated detection of retrieved diaspores was assessed by comparing the number of remaining diaspores manually counted on the videos every 30 minutes (*D*_*count*_) with the corresponding number (*D*_*track*_) obtained from image analysis (see Supplementary: S1 Automatic census). The agreement between these values was high, with both values being linearly related with a slope close to 1 (Fig. 5B), highlighting the robustness of our method.

Finally, to check the accuracy of the seeds detection, we compared manual and automated census of seeds and ants on a randomly located square portion of the arena (0.8% of the arena surface) and containing at least 1 seed. We obtained measurements on 37 frames randomly selected among all the replicates. This control showed that our method has a good detection rate of the seeds of 94.8% (55 seeds detected from a total of 58) associated with a strong discrimination ability with a specificity level of 100% (none of the 43 observed ants were mistaken for a seed).

### Colony activity on the arena during exploration and foraging stages

Hereafter, we use the term colony activity to refer to the number of ants detected in the arena during either exploration or foraging stages.

**Figure 6:**
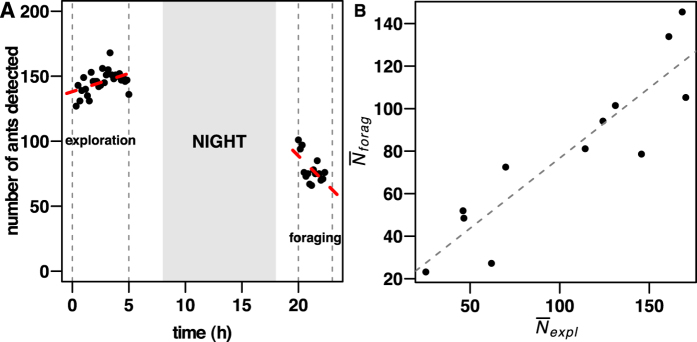
Colony activity as number of ants in the arena, at different stages of the experiments. (**A**) Variation through time of the number of ants in the arena during exploration and foraging stages (*N*_*explo*_ and *N*_*forag*_ respectively) for replicate number 2 that illustrates well the average dynamics observed (slight variation and significant decrease of ants number in the arena during exploration and foraging stages respectively). Red dotted lines stand for the linear regression (linear relationship: *N*_*explo*_ = 2.8*t* + 138.2, *R*^2^ = 0.19, *F*_1,27_ = 7.57, *P* = 0.01; *N*_*forag*_ = −8.7*t* + 91.6, *R*^2^ = 0.31, *F*_1,13_ = 7.31, *P* = 0.02). (**B**) Mean number of ants in the arena during exploration (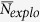) and foraging (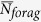) are linearly related (linear relationship: 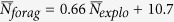, *R*^2^ = 0.80, *F*_1,10_ = 43.9, *P* < 0.0001). Each dot corresponds to a replicate.

During exploration stage, the number of workers (*N*_*explo*_) in the arena was independent of time in 50% of replicates (linear relationship: *N*_*explo*_ = *f(time*), *P* > 0.05; see Supplementary Table S4 for statistical tests), while in most of other replicates the number of workers increased up to 113 ± 119% (n = 6) of its initial value (Fig. 6A). On the contrary, there was a significant decrease of the amount of ants in the arena (*N*_*forag*_) throughout the foraging stage in 7/12 replicates (see Supplementary Table S4 for statistical tests), as much as −25.8 ± 5.3% of its initial value (Fig. 6A).

Interestingly, the mean colony activity during exploration and foraging stages were linearly related to each other (Fig. 6B). This characteristic level of activity of each colony was directly related to their size (exploration stage: 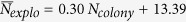, *R*^2^ = 0.43, *F*_1,10_ = 9.45, *P* = 0.01 ; foraging stage: 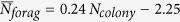, *R*^2^ = 0.70, *F*_1,10_ = 26.61, *P* = 0.0004).

### Diaspore retrieval on the platform

**Figure 7:**
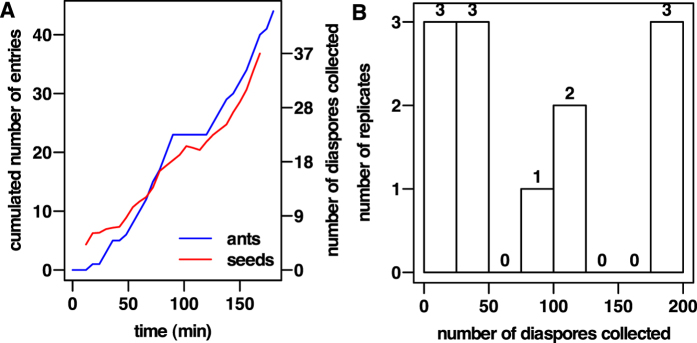
Diaspore collection was tightly related to the flow of ants entering the platform while its effectiveness was highly variable among replicates. (**A**) Time series of the cumulated number of ants having entered the platform and of the total amount of collected diaspores show a high correlation with each other (Spearman’s correlation test *ρ* = 0.99, replicate 2 depicted here). (**B**) Histogram of number of diaspores *D*_*retr*_ collected by each colony during the foraging stage (n = 12).

At the beginning of the experiment (Foraging stage), few workers entered the platform and a certain time was required for the diaspore retrieval process to start. The inflow of foragers entering the platform increased non-linearly during a period of time whose duration was specific to each replicate (Fig. 7A, blue line). Then, the inflow of ants remained stable over time. Similarly, once retrieval had started, its rate increased until it remained quite stable over time for each replicate (Fig. 7A, red line). This profile of foragers mobilization towards the platform suggests that recruitment phenomena and/or individual memory could be at work in the exploitation of the diaspore source.

There was a high correlation between the time series of the number of diaspores retrieved and the cumulated number of ants that had entered the platform (Spearman’s correlation test: *ρ* ≥ 0.96, n = 9; Fig. 7A), regardless of the replicate considered. The proportion of workers that reached the platform and went back to the nest loaded with a diaspore was high, with a mean individual collection ratio of *η*_*loaded*_ = 0.84 ± 0.11 diaspores/ant (n = 9; calculated as *η*_*loaded*_ = *D*_*retr*_/*N*_*ants*_, with *D*_*retr*_ the total number of collected diaspores and *N*_*ants*_ the total number of ants that entered the platform).

Concerning the global efficiency of retrieval, the number of retrieved diaspores (*D*_*retr*_) was highly variable, and in only three replicates all the available items (200) were collected during the foraging stage (Fig. 7B).

Surprisingly, neither the mean number of ants in the arena (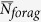) nor the proportion of colony population outside the nest (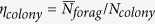) could be used to predict the total number of collected diaspores (*D*_*retr*_; linear relationships: 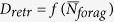, *P* = *NS*; *D*_*retr*_ = *f(η*_*colony*_), *P* = *NS*). Colony activity remained remarkably stable during exploration and foraging stages but could not be used to predict the number of diaspores that would be ultimately retrieved.

### Seed rejection

**Figure 8:**
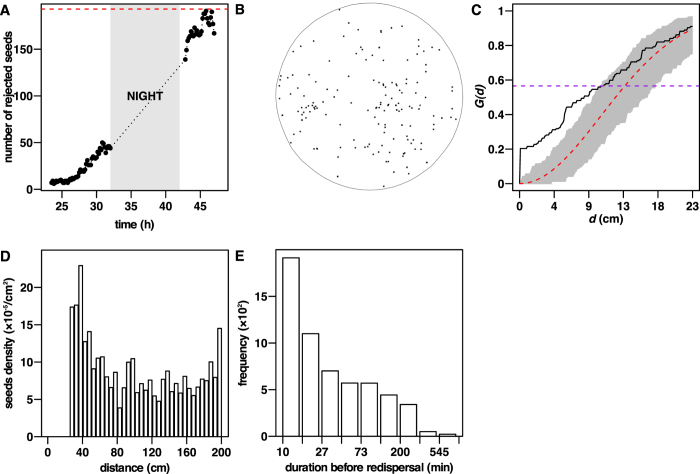
Dynamics of seed rejection from the nest, dispersal pattern and redispersal process (**A–C** depict results from replicate number 2 that illustrates well the average dynamics and spatial patterns observed; (**D**,**E** depict results of regrouped replicates). (**A**) Dynamics of seed rejection from the nest to the arena. Rejection occured even during the night. Red horizontal dashed line indicates the number of rejected seeds manually counted at the end of the experiment. (**B**) Final pattern of rejected seeds at the end of the experiment, on which clusters of seeds are visible. Gray circle depicts the area of interest used for the 1000 simulations of Complete Spatial Randomness (CSR). (**C**) Experimental *G(d*) function from observed pattern and pointwise envelope of 1000 simulated CSR. The *G(d*) function of observed pattern (black line) is above the pointwise envelope (grey shades). This departures from CSR is statistically significant (experimental line above the envelope: DCLF test *U* = 9.28, rank = 1, *P* = 0.001) and therefore indicates that the seeds pattern is clustered. The characteristic clustering distance is low (*NNdist*_*cluster*_ = 0.1 cm [0.1; 2.9], *n* = 56; from fraction of black curve below the purple dashed line) when compared to the higher characteristic CSR distance (*NNdist*_*CSR*_ = 6.7 cm [4.6; 8.5], *n* = 56000; fraction of envelope below the purple dashed line). (**D**) Density of seeds as a function of their distance to the centre of the nest, at the end of rejection stage. Seeds have been grouped as a function of their distance to the nest into 10 cm-wide concentric crowns centred on the nest. Data from all replicates are pooled. (**E**) Histogram (logarithmic abscissa) of the duration before redispersal of the seeds in the arena indicates that most of the seeds are quickly redispersed by workers.

Manual counting of the seeds in the arena at the end of the experiment (*S*_*count end*_) showed that most of the collected seeds were rejected from the nest to the arena at the end of the rejection stage, with a median rejection rate of 97%[95; 100] (n = 12; manual measurement). Complete rejection dynamics were obtained by automatic detection of the seeds: at first, seeds appeared slowly in the arena before reaching a higher and quite stable rejection rate (Fig. 8A). The mean fraction of seeds rejected hourly indicated that half of the seeds were rejected during the day (1 hour before the night: *η*_*rejected*_ = 0.44*S*_*count end*_ [0.31; 0.66], n = 12), nearly all of the remaining seeds being almost entirely rejected during the night (1 hour after the night: *η*_*rejected*_ = 0.93*S*_*count end*_ [0.84; 1.02], n = 12). In several replicates, total number of seeds detected at the end of the experiment (*S*_*track end*_) slightly exceeded the number counted by hand (*S*_*end count*_). This was due to the appearance of light stains over the arena, probably by ants excretions, which size and colour can be close to that of seeds. This error of estimation remained stable whatever the number of seeds rejected (linear relationship: *S*_*track end*_ = 0.86*S*_*count end*_ + 8.26, *R*^2^ = 0.98, *F*_1,10_ = 728, *P* < 0.0001).

We used circular statistics to analyze the distribution of seeds at the end of the experiments. For all trials, we found that the null hypothesis of uniformity could not be rejected (Rao’s spacing test, Kuiper test, Watson test, and Rayleigh test, *P* < 0.1). The same held for the circular distribution of all seeds cumulated in all experiments. Hence, the distribution pattern of seeds was not statistically different from an isotropic pattern: seeds were found in any direction with equal probability.

The spatial pattern analysis indicated that at the end of the experiments, 10 of the 12 replicates exhibited a clustered pattern (*P* ≤ 0.05; Fig. 8B and C), the 2 others not being statistically different from Complete Spatial Randomness (CSR). The fraction of seeds lying above the CSR pointwise envelope defines the characteristic clustering distance (*NNdist*_*cluster*_) which was low (*NNdist*_*clusert*_ = 4.6 cm [0.1; 9.2], *n* = 553; data pooled from each replicates), when compared to the characteristic CSR distance of the same fraction of seeds within the envelope (*NNdist*_*CSR*_ = 11.4 cm [5.6; 15.3], *n* = 553000; data pooled from each replicates simulation).

Hereafter, we pooled the values of all 12 replicates together. By the end of the experiments, half of the seeds were located at a median distance of 141 cm [93; 179] (n = 1007) from the nest. This median distance showed a low and non significant decrease through time (3.9 cm in 24 hours; linear relationship: *distance* = −0.164 *time* + 142.5, *R*^2^ = 0.03, *F*_1,82_ = 3.5, *P* < 0.065). There was a high proportion of seeds close to the arena wall, 10% being located at less than 5 cm from it. This was well observed on the radial pattern of rejected seeds: indeed, when considering the density of seeds per surface unit (cm^2^) as a function of the distance from the nest centre (cm), the density profile increased at its end instead of showing a flat tail (Fig. 8D). This indicates that these seeds located close to the arena wall could have been rejected even further than 2 meters away from the nest in a wider arena.

Finally, analysis of the seeds’ location within the arena showed that most of them stayed at a given position for only a short amount of time before being redispersed (median: 20 min [10; 70], *Q*_90_ = 170 min; Fig. 8E). The duration (*λ*) before seed redispersal was not related to their distance (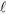) to the nest (linear relationship: 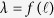, *R*^2^ = 0.005, *F*_1,5709_ = 30.3, *P* = 0.0009).

We computed the minimum redispersal distance as the distance between any seed last location before its redispersal and the location of the closest seed among those newly appeared within 20 minutes (2 images) after redispersal. The median distance of redispersal was 6.3 cm [1.5; 19.5] (n = 4420). Such value seems too large to be the result of sole accidental bumping between ants and seeds, but suggests on the contrary that seed redispersal is the result of an active transportation by ants.

Our analysis showed that seed dispersal exhibits the same centrifugal dispersal nature than other waste release processes (excavation refuses^67^, corpses^68^). They also share in common the random orientation of ants when leaving the nest, explaining the isotropic nature of the seeds pattern. We thus performed simulations to determine how far simple isotropic centrifugal dispersal^67^ could lead to seeds clustering (see Supplementary). Our simulations indicated that a model with “blind” seeds dropping (dropping independently of previously deposited seeds) rarely produced clustered patterns, while “reactive” seeds dropping (dropping dependent of the encounter of previously deposited seeds within a short perception range of 5 mm) generated clustered patterns in most of the cases (see Supplementary and Fig. S4). The reactive dropping favoured the formation of seeds clusters, however these clusters were on average closer to the nest than observed in our experiments.

## Discussion

The study of seed dispersal by animals has always been challenging, mainly due to difficulties of following dispersers and their loads. Myrmecochory provides an additional challenge, due to the small size of both actors (ants and seeds). As a result, this study introduces and validates a semi-automatic methodology to study key steps of myrmecochory under laboratory conditions, notably the rejection stage of seeds and their subsequent redispersal process, about which knowledge remains scarce.

From a biological perspective, we found no link between colony activity and the efficiency of diaspore collection (expressed as the number of collected diaspores on the foraging platform). Similarly, Kjellsson and colleagues^11^ found no link between the foraging activity of *Myrmica ruginodis* and the number of ants actively foraging on diaspores of *Carex pilulifera*. Because the quantity of larvae and queens was not controlled in our experiments, it is possible that variability in the number of diaspores retrieved is linked to the differential nutritional status of the tested colonies. Yet, our experiments indicate that colony activity is not a reliable predictor for estimating efficiency in the exploitation of the source of diaspores. One hypothesis is that workers inside the nest represent an important part of the ants involved in the exploitation of the diaspore source. The inflow of workers towards the foraging platform showed a non-linear profile at the beginning of the experiment. Thus, diaspore exploitation might be based on individual memory and/or non-directional recruitment. Indeed, even though we did not observe any recruitment similar to the one that occurred during the foraging of sucrose solution, the diaspores brought back to the nest might have enhanced the motivation of inner nest foragers or workers to go outside^11^, triggering non-directional recruitment. These two mechanisms - memory and/or the stimulation of ants inside the nest – need to be tested through theoretical studies to identify key behaviours and to simulate the observed foraging dynamics. Moreover, the removal rate of diaspores is linked to the experience of workers^45^,^69^. Future investigations should focus on this aspect by repeated observations (i.e. several trials) of seed foraging in the same colony for which the nutritional state is controlled precisely.

Most diaspores (98%) brought into the nest during the foraging stage were rejected within 24 h and exhibited a clustered pattern. All seeds that were rejected no longer had their elaiosome, suggesting that the ants had consumed this part of the diaspore. These results are consistent with those of other studies on seed rejection^7^,^34^,^51^. Of note, Canner and colleagues^51^ found that a lethal fungus grew on seeds that remained in the nest under laboratory conditions. Seeds lacking elaiosomes could simply be considered as a waste, and be removed as part of prophylactic nest cleaning behaviour. Hence, very few seeds remained inside the nest at the end of our experiments, with most seeds being rejected within one day. Moreover, once in the arena, many rejected seeds were subject to recurrent redispersal, the frequency of which was not related to their proximity to the nest. The amplitude of the minimal redispersal distance suggests that this secondary dispersal of the seeds in the arena is not due to accidental collision between ants and seeds but is rather due to an active transportation by the workers. Such redundant redispersal process was not expected because seeds deprived of their elaiosomes are supposed to no longer be attractive to ants.

In our simulations, we purposely omitted any redispersal components since we did not observe these behaviours in our experiments. The different distances of seed clusters to the nest between simulations and experiments suggest that redispersal may also play a role in the clustering process. Seed dispersal may in fact be a combination of isotropic centrifugal rejection similar to that observed with various other items (soil particles^67^, corpses^68^) and of secondary dispersal and clustering of previously released seeds with behaviours analogous to those at work during the formation of cemeteries^70^. This particular point would require new dedicated experiments to quantify the redispersal process and to identify the individual behaviours involved.

Therefore, this final step of the myrmecochorous process of dispersal might be much more continuous and dynamic than previously expected, with similar complexity occurring for the many other items that ants are known to manipulate, such as corpses, soil grains or larvae, for which they form various spatio-temporal patterns, such as cemeteries, nest mounds, and brood clusters, by spatial rearrangement^67^.

This redispersal process has a major impact on the ultimate benefits received by the plant in this partnership, by influencing inter-seed distance and the final distance of seeds from the parent plant, which might act positively or negatively on fitness. Further analysis of the observed spatio-temporal pattern of seeds within the arena would help improve our understanding on the implications of secondary dispersal in the myrmecochory process and, thus, the mutualistic relationship at work here.

Our method uses low budget materials and USE Tracker free software; however, it allowed us to detect almost 95% of seeds in each picture, despite seeds covering just a few pixels of the surface area of very large pictures, and despite noise and illumination variability in images. We were also able to reliably estimate the number of ants exploring the arena. Because ants are very mobile in comparison to seeds, we were also able to distinguish and discriminate seeds from ants during the removal stage.

Several benefits directly stem from our method. For instance, only a computer and 2 DSLR cameras are required to produce large pictures to observe the seeds at a relatively low cost. Also, the different image processing algorithms allowed us detect a large number of seeds in very short time. Thus, we were able to observe the evolution of seed distribution during the course of the experiments, rather than being restricted to the end of the dispersal, as in previous works^48^,^51^. Finally, because data acquisition is automated, no intervention is required, other than the parametrization of the image processing algorithms, and the correction of systematic errors which can be quantified. We assessed this bias, as well as the reliability and robustness of our detection algorithms, through several validation steps. We compared the results of manually counted items versus automated detection, and found good agreement between these two methods; thus, validating that the image analysis procedure is effective at detecting and discriminating items. Moreover, our analysis showed that using the same set of parameters to analyse all of our experiments led to the effective and stable detection of ants through time and between replicates, which allowed the reliable pooling of data. Our experimental setup allows us to make very precise observations with fine control over the conditions. Simple improvements of this initial framework would greatly enhance our understanding of seed dispersal. For instance, an increased sampling rate (e.g. a picture every minute) would allow us to estimate the minimum duration before redispersal with greater accuracy. Night-time activity could also be observed using infrared cameras, with minimal changes to the current pipeline of image processing algorithms. This addition would provide a finer understanding of seed rejection dynamics. However, the characteristics of the arena, which is flat and devoid of any obstacles between the nest and the borders, might overemphasise the rate of seed redispersal. For instance, while it is clear that redispersal is an important aspect that has been previously overlooked, our specific laboratory settings might increase the probability for ants to pick them up, due to seeds lying in the open.

Our method could be easily transposed to several situations in which entities (transported items, animals) move over a large distance in comparison to their size. Our freely available software USE Tracker is versatile enough to address the requirements of numerous conditions. This program accepts different inputs, such as still pictures or webcam videostreams, depending on the desired temporal and spatial resolution. In addition, to cover wider areas, multiple inputs can be stitched together. The modular architecture of the software allows several different algorithms to be combined to grasp the particular characteristics of specific entities within their surroundings. Finally, the USE Tracker graphical interface allows real-time fine-tuning of the image analysis parameters, even at the stage of experimental setup implementation, to achieve optimal image capturing and data processing.

## Additional Information

**How to cite this article**: Bologna, A. *et al*. An automated method for large-scale monitoring of seed dispersal by ants. *Sci. Rep.* **7**, 40143; doi: 10.1038/srep40143 (2017).

**Publisher's note:** Springer Nature remains neutral with regard to jurisdictional claims in published maps and institutional affiliations.

## Supplementary Material

Supplementary Information
