# Supplementary material for: An automated method for large-scale monitoring of seed dispersal by ants
Source: Sci Rep. 2017 Jan 10;7:40143. doi: 10.1038/srep40143 (PMC5223120; doi:10.1038/srep40143)
Supplement: Supplementary Information [file srep40143-s1.pdf]

An automated method for large-scale monitoring of seed dispersal by ants. Supplementary file

Audrey Bologna[1,*,+], Etienne Toffin[1,+], Claire Detrain[1], and Alexandre Campo[1]

[1]Unit of Social Ecology, Université Libre de Bruxelles, Campus de la Plaine, Brussels, Belgium
[*]aubologn@ulb.ac.be
[+]these authors contributed equally to this work

S1. Automatic census of remaining diaspores on the platform

Initial processing of the data

Using USE Tracker software, diaspores and ants were indistinctly detected by background subtraction algorithm on each frames of the 3 hours movies of the platform (Figure S1 B). Numerical output for each frame of the movie consisted of the total number of pixels detected, corresponding to both diaspores and ants. Values collected on each frame were aggregated on a 10 seconds basis (*i.e.* total number of pixels detected in 50 consecutive frames, movies being at 5 frames/s; Figure S2 **B**), before computing the mean number of detected pixels $D_{track}(t)$ using a rolling mean of window $k = 300$ s (Figure S2 **B**).

The detected pixels correspond to both diaspores and ants as

$$Pix(t) = D_{pix}(t) + A_{pix}(t) \tag{S1}$$

where $D_{pix}(t)$ and $A_{pix}(t)$ are the number of pixels corresponding to the diaspores and the ants respectively. Computing the number of diaspores remaining on the platform $Dp(t)$ is then executed in two steps:

1. determine the number of ants on the platform
2. determine the relative size of diaspores and ants

Number of ants on the platform

The estimated number of ants located on the platform at each time step $A_{est}(t)$ (Figure S2 **A**) was determined by using the timing of entrances and exits of workers (data of incoming flow and outgoing flows collected by hand with USE Tracker). The accuracy of $A_{est}(t)$ was then assessed by counting the number of workers located on the platform $A_{count}(t)$ at different time steps $t \in [30; 60; 90; 120; 150; 180]$ minutes. In 6 of 9 replicates, the estimated number of ants $A_{est}(t)$ at t=180 min was higher than the number obtained by a manual counting as $A_{est}(t) = A_{count}(t) + \Delta$, with $\Delta > 1$ ant (Figure S2 **A**).

The error Δ was due to ants leaving the platform unnoticed, by falling or exiting the platform while out of sight (*e.g.* walking on lower face of the access ramp). Since Δ was linearly related to the total number of ants A_{total} that had entered the platform at the end of the foraging phase ($\Delta = 0.080\,A_{total} - 1.48$, $R^2 = 0.96$, $F_{1,7} = 203.1$, $P < 0.0001$), we computed a corrected value of the number of ants on the platform $A_{corr}(t)$ (Figure S2 **A**) from $A_{est}(t)$ by subtracting an amount of ants (*i.e.* those leaving the platform unnoticed) proportional to a constant fraction Ψ of the cumulated number of workers $A_{cum}(t)$ that had entered the platform at time t as $A_{corr}(t) = A_{est}(t) - \Psi\, A_{cum}(t)$.

The value of Ψ was different for each replicate and has been determined by optimisation, so as to minimize the difference between $A_{count}(t)$ and $A_{corr}(t)$ computed as

$$RSS = \sum_{t=30}^{180} (A_{corr}(t) - A_{count}(t))^2 \tag{S2}$$

with $t \in [30; 60; 90; 120; 150; 180]$. The values of Ψ ranged from 0.04 to 0.085 (n=6 replicates). The correction with Ψ allowed a reduction of the values of RSS computed with A_{corr} ranging from 60% to 95% compared (n=6 replicates) to that obtained with $A_{est}(t)$ (*i.e.* $\sum_{t=30}^{180}(A_{est}(t) - A_{count}(t))^2$); Figure S2 **A**). After this step, the number of ants on the platform was available to compute the remaining number of diaspores.

Number of diaspores on the platform

The mean number of pixels δ corresponding to a diaspore was computed at t=0 when only the 200 diaspores were on the platform. By pooling the values of pixels $Pix(t)$, counted diaspores $D_{count}(t)$ and ants $A_{count}(t)$ from each replicate at 6 different times $t \in [30; 60; 90; 120; 150; 180]$ minutes, we determined the ratio ρ between diaspores and ants size using the following linear relationship $Pix(t)/\delta - D_{count}(t) = \rho\, A_{count}(t)$ (linear regression: $\rho = 3.49$, $R^2 = 0.94$, $F_{1,53} = 914.4$, $P < 0.0001$). Finally, the number of diaspores $Dp(t)$ on the platform was computed as $Dp(t) = Pix(t)/\delta - \rho\, A_{corr}(t)$.

As a last step, the number of diaspores on the platform was smoothed using a rolling mean of window $k = 15$ minutes, and the resulting value $D_{track}(t)$ was used in the analysis.

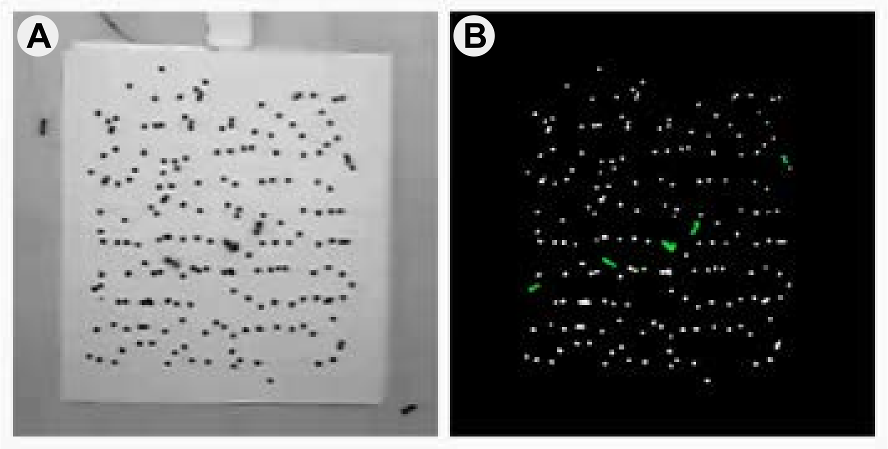

Figure S1. Diaspores and ants on the platform are automatically detected by USE Tracker. **A** Input frame. **B** Output picture. The value $Pix(t)$ used in our analysis is the number of white pixels which represent diaspores and ants.

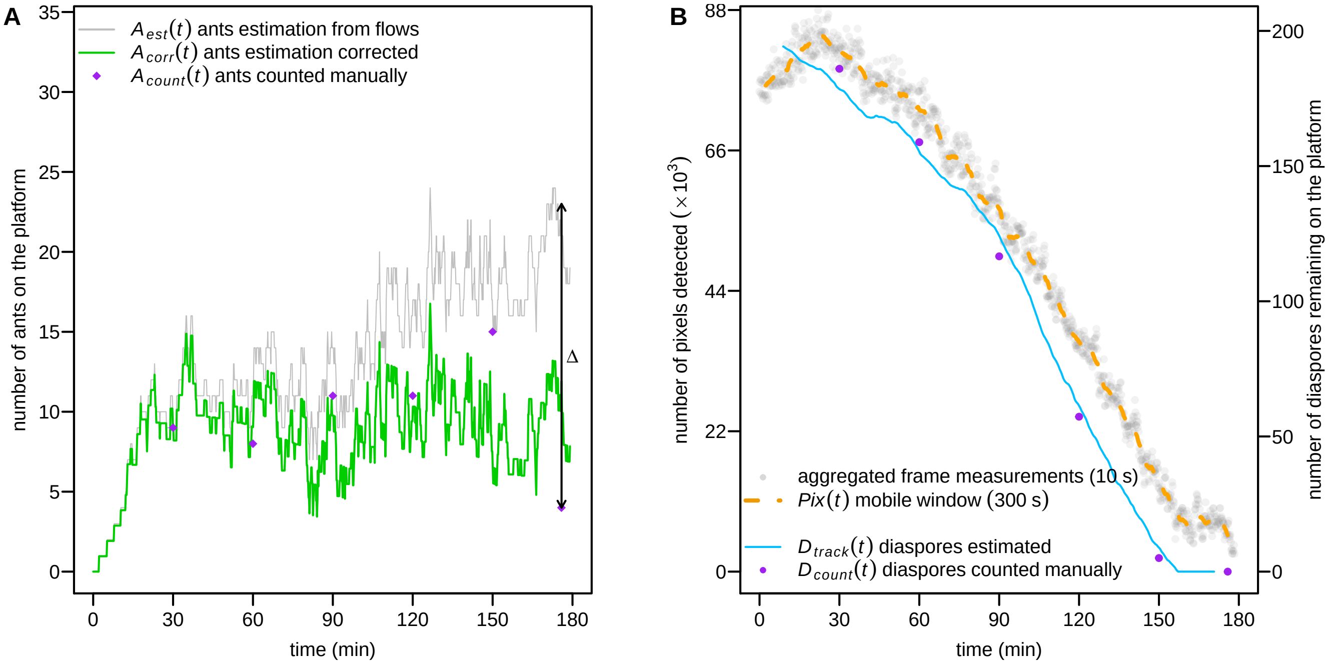

Figure S2. Estimation of (**A**) ants $A_{corr}(t)$ and (**B**) diaspores $D_{track}(t)$ on the platform. **A** Number of ants $A_{est}(t)$ predicted by incoming and outgoing ants flows shows an important error Δ at the end of the experiment, which is corrected $A_{corr}(t)$ by constantly subtracting a fraction Ψ of the total number of ants $A_{cum}(t)$ that have reached the platform. **B** Number of pixels detected by the tracker was aggregated (sum) over a 10 s duration, and smoothed with a rolling mean of window $k = 300$ s ($Pix(t)$). Estimate of the number of diaspores remaining on the platform $D_{track}(t)$ was computed before comparison with manually counted numbers D_{count}.

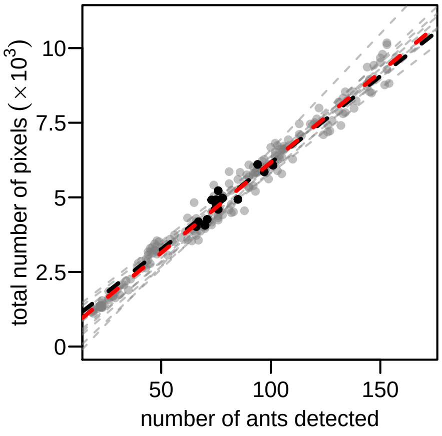

Figure S3. During the foraging stage, effectiveness of ants detection was relatively stable since relationship between number of ants detected and the total number of corresponding pixels was similar between replicates (see linear regression values in Table S3). Black dots and black dotted lines stand for values and linear regression of replicate 5, while red dotted lines indicates global regression line for all replicates.

S2. Simulations of seed dispersal

Model

Our analysis indicates that the seeds are released isotropically within the arena (see Seed rejection, in Results). This finding is in agreement with dispersal of other wastes away of the nest, such as soil particles during nest excavation[1] and corpses disposal.[2] The results also show the importance of redispersal all along the rejection stage, but these findings were unexpected and our data do not contain any quantification about the individual behaviours involved.

We thus model the seeds rejection and purposely ignoring redispersal behaviours (catch/drop of previously released seeds), to investigate the ability of the model of craters building through soil rejection[1] to generate clustered patterns. The procedure consists of centrifugal seeds dispersal, isotropically distributed around the nest (seeds are expelled from nest in random directions, following straight lines). Two alternate models based on this procedure are investigated :

1. blind drop model. Ants follow a straight exit trajectory from the nest, seeds dropping is independent from previous deposits

2. reactive drop model. Ants follow a straight exit trajectory from the nest, seeds dropping is dependent of previous deposits within a short scale (5 mm): when encountering seeds within this scale, ants drop their load in the vicinity of this previous deposit (1 mm apart).

In each simulations, 200 seeds are released from the nest located at (0;0), into the simulated arena of 2000 mm radius. Each seed is released along a straight trajectory oriented at a random angle θ with $\theta \in [-\pi;\pi[$, and drop away from the nest at a distance ℓ (cm) sampled from the experimental density function of seed distance to the nest. The final spatial pattern is characterized using $G(d)$ function and DCLF test (see Data analysis, in Material and Methods section). We realize 1000 simulations for each model with the same number of seeds (200) to be released.

Simulation results

The simulations indicate that the blind drop model produces clustered pattern in only 5.9% of the simulations (59 of 1000 simulations). On the contrary, the reactive model produces clustered patterns in 97.9% of the simulations (977 of 1000 simulations), what is much closer to the ratio of clustered pattern observed in the experiments (10 of 12 replicates).

The distribution of the nearest neighbor distance reflects these results: experience as well as reactive drop model produce a significant fraction of short distances (between 10 and 30% of all distances are below 1 mm); blind drop model on the contrary exhibits a more uniform distribution of distances (Figure S4 **B** & **C**).

If the blind model shows radial densities of seeds in agreement with the one observed in the experiments (as expected since simulated rejection distances follow the empirical distribution of distances), on the contrary the reactive model exhibits more seeds between 25 and 40 cm from the nest (Figure S4 **A**). This suggests that most of the clustering in the reactive model occurs in this region (median distance to the nest: experiments=146.8 cm [95.5;183.8]; blind=140.9 cm [92.9;178.4]; reactive=133.4 cm [77.8;174.4]).

This indicates that the reactive model partially reproduces the observed patterns: it is able to generate clustered patterns, but the location of the clusters is not in complete agreement with the experimental patterns. We hypothesize that the redispersal behaviours could play a significantly role in the clustering process and its absence in our model could explain the partial disagreement between our experiments and the simulations.

References

1. Theraulaz, G., Gautrais, J., Camazine, S. & Deneubourg, J.-L. The formation of spatial patterns in social insects: from simple behaviours to complex structures. *Phil Trans R Soc Lond A* **361**, 1263–1282, DOI:10.1098/rsta.2003.1198 (2003).

2. Diez, L., Deneubourg, J.-L. & Detrain, C. Social prophylaxis through distant corpse removal in ants. *Naturwissenschaften* **99**, 833–842, DOI:10.1007/s00114-012-0965-6 (2012).

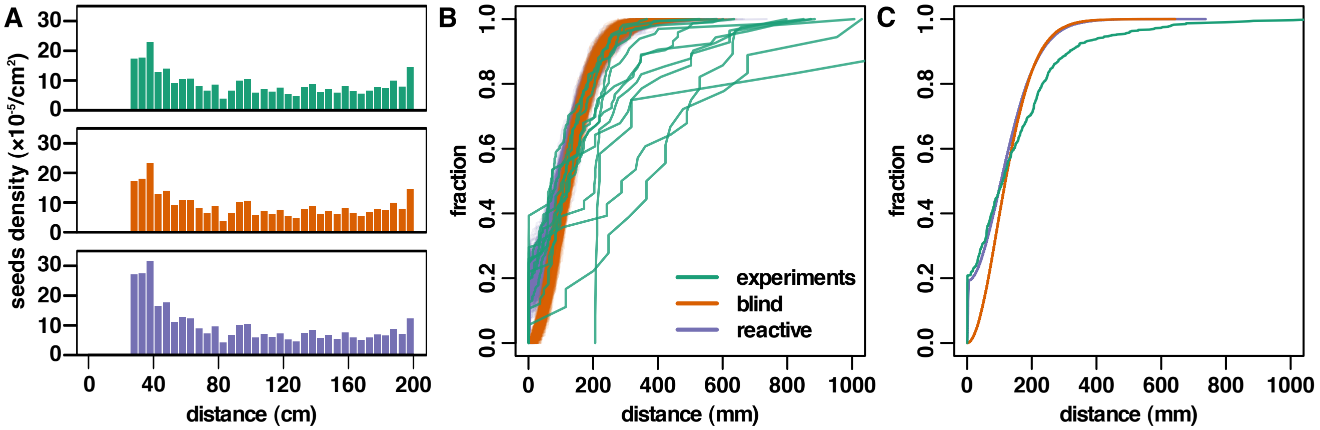

Figure S4. **A** Density of seeds as a function of their distance to the centre of the nest, at the end of the rejection stage. Reactive model (purple) exhibits highest density of seeds within the 25-40 cm range, than those observed in experiments (green) and blind model (orange), these last two showing similar density of seeds as a function of distance to the nest. Seeds have been grouped as a function of their distance to the nest into 10 cm-wide concentric crowns centred on the nest. Data from all replicates and simulations are pooled by condition. **B-C** Cumulative distribution of nearest neighbor distances. Similarly to experiments, reactive model shows a large fraction of short nearest-neighbour distances (ca. 1 mm), what is not the case of the blind model. **B** Individual curves (each replicate is depicted by a single line). **C** Condition curves (each line represents all the regrouped distances from a condition).

	Auto (tracker)	Manual
Foraging platform	- #pixels on platform (continuous)<br>$\rightarrow D_{track}$ = #remaining seeds on platform (see Supp 1)	- D_{count} = #remaining seeds on platform (each 30 min)<br>- #ants entering/leaving platform (continuous)<br>- A_{count} = #ants on platform (each 30 min)
Arena	- X,Y coordinates & size of ants (each picture, 10 min)<br>$\rightarrow N_{explo}$ = #ants during exploration<br>$\rightarrow N_{forag}$ = #ants during foraging<br>- X,Y coordinates & size of seeds (each picture, 10 min)<br>$\rightarrow S_{track}$ = #seeds<br>$\rightarrow \theta$ = angle within arena<br>$\rightarrow \ell$ = distance from nest<br>$\rightarrow nnDist$ = seed-to-nearest-seed distances	- $S_{end\ count}$ = #seeds rejected at end (last picture)

Table S1. Summary of raw extracted data and variables used in the analysis. Raw collected data are sorted by location (Foraging platform/Arena; rows) and collection method (Automatic/Manual; lines), and the variables extracted from them are detailed after the arrow $\rightarrow$. Measurement interval is given in parenthesis.

	Estimate	Std. Error	t value	Pr(>|t|)
(Intercept)	-1735.6365	1104.1173	-1.57	0.1169
numBlobs	73.4863	6.5428	11.23	0.0000
factor(replicate)1	2162.7431	1485.8061	1.46	0.1465
factor(replicate)2	1466.1193	1693.8579	0.87	0.3874
factor(replicate)3	-1093.5235	1348.7281	-0.81	0.4181
factor(replicate)4	1413.6053	1334.4799	1.06	0.2903
factor(replicate)5	698.8551	1227.3465	0.57	0.5695
factor(replicate)6	1792.8696	1329.0904	1.35	0.1783
factor(replicate)8	-1265.9913	1408.3248	-0.90	0.3694
factor(replicate)9	2080.9661	1272.1200	1.64	0.1029
factor(replicate)10	1703.8838	1177.6892	1.45	0.1489
factor(replicate)11	1973.1455	1331.6765	1.48	0.1394
factor(replicate)12	-1734.3159	1683.2084	-1.03	0.3036
numBlobs:factor(replicate)1	19.6627	8.9881	2.19	0.0294
numBlobs:factor(replicate)2	4.9111	10.9713	0.45	0.6547
numBlobs:factor(replicate)3	5.3124	7.9561	0.67	0.5048
numBlobs:factor(replicate)4	-7.7337	12.5266	-0.62	0.5374
numBlobs:factor(replicate)5	-1.4447	8.0264	-0.18	0.8573
numBlobs:factor(replicate)6	3.6829	17.1260	0.22	0.8299
numBlobs:factor(replicate)8	13.5040	9.3266	1.45	0.1486
numBlobs:factor(replicate)9	-8.7623	12.0667	-0.73	0.4683
numBlobs:factor(replicate)10	-3.2068	17.2382	-0.19	0.8525
numBlobs:factor(replicate)11	-16.7937	17.3701	-0.97	0.3344
numBlobs:factor(replicate)12	23.9695	12.1327	1.98	0.0491

Table S2. Linear regression analysis of detected blobs against number of pixels during exploration stage, using replicate as factor on both intercept and slope. Analysis indicates that considering a complete model (a common slope *numBlobs* for the entire set of values, all replicates regrouped) is statistically significant, while considering independent slopes for each replicate ($factor(replicate)n$) does not add statistically significant decrease of the residuals compared to complete model.

	Estimate	Std. Error	t value	Pr(>|t|)
(Intercept)	-405.5590	993.2703	-0.41	0.6835
numBlobs	65.2174	6.8119	9.57	0.0000
factor(replicate)1	147.1877	1351.9653	0.11	0.9134
factor(replicate)2	763.3025	1121.7142	0.68	0.4971
factor(replicate)3	403.2601	1102.9159	0.37	0.7151
factor(replicate)4	-772.2326	1225.0561	-0.63	0.5292
factor(replicate)5	-66.0166	1092.5386	-0.06	0.9519
factor(replicate)6	986.9166	1126.4088	0.88	0.3821
factor(replicate)8	-482.4931	1236.7937	-0.39	0.6969
factor(replicate)9	536.1132	1053.1803	0.51	0.6113
factor(replicate)10	391.7799	1067.7024	0.37	0.7141
factor(replicate)11	740.1089	1057.7388	0.70	0.4850
factor(replicate)12	1085.1923	1118.3804	0.97	0.3332
numBlobs:factor(replicate)1	-3.8715	9.6494	-0.40	0.6887
numBlobs:factor(replicate)2	-7.2006	9.4665	-0.76	0.4479
numBlobs:factor(replicate)3	-1.7819	8.1721	-0.22	0.8276
numBlobs:factor(replicate)4	12.5031	11.9783	1.04	0.2980
numBlobs:factor(replicate)5	-2.1350	8.7909	-0.24	0.8084
numBlobs:factor(replicate)6	-8.7224	12.8360	-0.68	0.4977
numBlobs:factor(replicate)8	4.5920	9.9395	0.46	0.6446
numBlobs:factor(replicate)9	-8.4112	14.3727	-0.59	0.5591
numBlobs:factor(replicate)10	-3.0207	18.0264	-0.17	0.8671
numBlobs:factor(replicate)11	-6.4665	9.6932	-0.67	0.5055
numBlobs:factor(replicate)12	-8.2941	8.7040	-0.95	0.3419

Table S3. Linear regression analysis of detected blobs against number of pixels during foraging stage, using replicate as factor on both intercept and slope. Analysis indicates that considering a complete model (a common slope *numBlobs* for the entire set of values, all replicates regrouped) is statistically significant, while considering independent slopes for each replicate ($factor(replicate)n$) does not add statistically significant decrease of the residuals compared to complete model.

	exploration				foraging			
replicate	slope	intercept	R^2	Pr(>|t|)	slope	intercept	R^2	Pr(>|t|)
1	1.33	157.36	-0.01	0.42	-4.72	143.72	0.15	0.06
2	2.80	138.16	0.19	0.01	-8.68	91.62	0.31	0.02
3	9.29	145.58	0.62	0.00	-13.13	127.12	0.54	0.00
4	1.55	65.70	0.07	0.10	-3.03	77.80	0.12	0.09
5	6.53	96.80	0.31	0.00	-5.44	93.34	0.12	0.08
6	0.19	46.02	-0.03	0.77	-4.19	55.46	0.29	0.01
7	3.71	158.48	0.18	0.01	-9.17	159.22	0.39	0.01
8	1.97	125.83	0.03	0.19	-2.50	105.82	0.00	0.32
9	-2.37	68.21	0.18	0.01	-3.11	32.66	0.25	0.02
10	0.59	23.69	-0.00	0.35	-0.69	24.37	-0.04	0.53
11	0.57	44.54	-0.01	0.38	-6.59	63.48	0.36	0.01
12	2.08	118.58	0.13	0.03	-7.65	106.86	0.23	0.03

Table S4. Results of linear regression between time and number of ants in the arena during exploration and foraging stages. Statistical significancy of F-test is indicated (Pr(>|t|) as well as value of R^2.
